# Ascending the “Hill” of Artificial Intelligence in Upper Gastrointestinal Endoscopy

**DOI:** 10.1002/ueg2.12730

**Published:** 2024-12-12

**Authors:** Cem Simsek, Nasim Parsa, Lumir Kunovsky

**Affiliations:** ^1^ Division of Gastroenterology Hacettepe University Faculty of Medicine Ankara Turkey; ^2^ Division of Gastroenterology Hepatology and Nutrition University of Minnesota Medical Center Minneapolis Minnesota USA; ^3^ Satisfai Health Inc Vancouver Canada; ^4^ 2nd Department of Internal Medicine – Gastroenterology and Geriatrics University Hospital Olomouc Faculty of Medicine and Dentistry Palacky University Olomouc Olomouc Czech Republic; ^5^ Department of Surgery University Hospital Brno Faculty of Medicine Masaryk University Brno Czech Republic; ^6^ Department of Gastroenterology and Digestive Endoscopy Masaryk Memorial Cancer Institute Brno Czech Republic

**Keywords:** Barrett's esophagus, gastroesophageal junction, gastroesophageal reflux disease, hiatal hernia, large language models

The recent study by Kafetzis et al. on AI‐assisted Hill classification represents a significant advancement in the standardization of gastroesophageal junction (GEJ) assessment [1]. The importance of accurate GEJ evaluation cannot be overstated, particularly in the context of gastroesophageal reflux disease (GERD) and Barrett's esophagus. The Hill grade classification, an endoscopic classification of the anti‐reflux barrier focused on the flap valve, has been reported to correlate with the severity of GERD. However, clinical utilization of this classification has been limited mainly due to inter‐observer variability [2, 3]. The application of artificial intelligence (AI) in gastrointestinal endoscopy has gained significant traction in the past decade. Particularly in the field of upper gastrointestinal endoscopy, the application of AI has been focused on the detection and characterization of pre‐malignant and malignant lesions, and this work thus represents a part of the recent shift toward addressing this imbalance [4, 5, 6].

As we evaluate this study and contemplate future directions, it is crucial to consider the recent guidelines set forth by QUAIDE (Quality Assessment of pre‐clinical AI studies in Diagnostic Endoscopy) [7]. This framework provides valuable guidance for the design, reporting, and interpretation of AI studies in gastrointestinal endoscopy. The work by Kafetzis et al. aligns well with several QUAIDE recommendations, particularly in its patient‐based selection for test sets and multi‐annotator approach. The real‐time implementation and explainability features of their AI system are particularly commendable. These aspects not only enhance the system's potential for clinical integration but also address the need for transparency in AI‐assisted medical decision‐making.

Looking beyond the immediate implications of this study, we can envision a future where AI‐assisted Hill classification serves as a foundation for a comprehensive GEJ assessment. The integration of endoscopic imaging with the data from the high‐resolution esophageal manometry, PH studies, and EndoFlip could potentially provide a multidimensional view of GEJ function. Furthermore, the combination of Computer Vision with symptom analysis Large Language Models (LLMs) presents an exciting opportunity to bridge the gap between endoscopic findings and patient‐reported outcomes, aligning with the growing emphasis on patient‐centered care in gastroenterology [8]. The potential for AI to predict outcomes of anti‐reflux interventions is another avenue worthy of exploration. By synthesizing Hill grade classification with other patient‐related clinical parameters, AI could potentially optimize patient selection in an attempt to improve clinical success rates and patient outcomes [3].

As we contemplate these possibilities, it is crucial to recognize the importance of clinician involvement in AI development. Physician‐led initiatives, such as this study, supported by appropriate regulatory frameworks and funding, are essential to ensure that AI systems remain clinically relevant and easily adoptable. The single‐center design of the study underscores the necessity for multi‐institutional validation to ensure generalizability across diverse clinical settings. In this context, federated learning emerges as a promising approach to facilitate large‐scale, multi‐institutional studies while navigating the complexities of data privacy in healthcare. This approach could be particularly valuable in addressing the challenges of data sharing and collaboration [9].

The integration of AI‐assisted Hill classification into clinical practice could have profound implications for quality assurance and training in endoscopy. By providing objective, standardized assessments, it could serve as a valuable quality metric and revolutionize the training of new endoscopists [10]. In conclusion, the work by Kafetzis et al. represents not just an advancement in GEJ assessment, but a steppingstone toward a new paradigm in gastroenterology. It invites us to envision a future where AI‐enhanced, multimodal approaches lead to more precise, personalized, and effective patient care. As we pursue this vision, close collaboration between clinicians, researchers, and technologists will be crucial in realizing the full potential of AI in our field.

## Conflicts of Interest

Nasim Parsa: Speaker for Phathom Pharmaceuticals, VP Medical Affairs at Satisfai Health Inc. Cem Simcek and Lumir Kunovsky have no disclosures.

## Data Availability

Data sharing is not applicable to this article as no new data were created or analyzed in this study.
